# Advanced ultrasound methods in assessment of carotid plaque instability: a prospective multimodal study

**DOI:** 10.1186/s12883-020-1620-z

**Published:** 2020-01-29

**Authors:** M. Zamani, K. Skagen, H. Scott, D. Russell, M. Skjelland

**Affiliations:** 1 Department of Neurology, Oslo University Hospital, Rikshospitalet, Postboks 4950 Nydalen 0424 Oslo, Norway; 2 Department of Clinical Medicine, University of Oslo, Oslo, Norway; 3 Department of Pathology, Oslo University Hospital, Rikshospitalet, Norway

**Keywords:** Superb microvascular imaging, Unstable carotid plaque, Carotid plaque -neovascularization, Vulnerable plaque imaging, Shear wave Elastography, Contrast enhanced ultrasound, Atherosclerosis and ischemic stroke

## Abstract

**Background:**

A significant proportion of ischemic strokes are caused by emboli from atherosclerotic, unstable carotid artery plaques. The selection of patients for endarterectomy in current clinical practice is primarily based on the degree of carotid artery stenosis and clinical symptoms. However, the content of the plaque is known to be more important for stroke risk. Intraplaque neovascularization (IPN) has recently emerged as a possible surrogate marker for plaque instability. Neo-microvessels from the adventitial vasa vasorum grow into the full thickness of the vessel wall in an adaptive response to hypoxia, causing subsequent intraplaque haemorrhage and plaque rupture. Conventional ultrasound cannot detect IPN. Contrast-enhanced ultrasound and Superb Microvascular Imaging (SMI), have, however, shown promise in IPN assessment. Recent research using Shear Wave Elastography (SWE) has also reported reduced tissue stiffness in the artery wall (reduced mean Young’s modulus) in unstable compared to stable plaques. The purpose of this study is to identify unstable carotid artery plaques at risk of rupture and future ischemic stroke risk using multimodal assessments.

**Methods:**

Forty five symptomatic and 45 asymptomatic patients > 18 years, with > 50% carotid stenosis referred to Oslo University Hospital ultrasound lab will be included in this on-going project. Patients will undergo contrast enhanced ultrasound, SMI, carotid-MRI and PET-(^18^F-FDG). Contrast enhanced ultrasound will be analyzed semi-quantitatively (5-levels visual classification) and quantitatively by plotting time-intensity curve analyses to obtain plaque peak contrast enhancement intensity. Plaques removed at carotid endarterectomy will be assessed histologically and the number of microvessels, areas of inflammation, granulation, calcification, lipid and fibrosis will be measured.

**Discussion:**

This multimodality study will primarily provide information on the clinical value of advanced ultrasound methods (SMI, SWE) for the detection of unstable carotid artery plaque in comparison with other methods including contrast-enhanced ultrasound, carotid-MRI and PET-(^18^F-FDG) using histology as the gold standard. Secondly, findings from the methods mentioned above will be related to cerebrovascular symptoms, blood tests (leukocytes, CRP, ESR, lipoproteins and inflammatory markers) and cardiovascular risk factors at inclusion and at 1-year follow-up. The overall aim is to optimize detection of plaque instability which can lead to better preventive decisions and reduced stroke rate.

## Background

Stroke is the 3rd leading cause of death and the most common cause of disability worldwide [1]. Thromboembolism from an unstable atherosclerotic plaque at the carotid bifurcation or the internal carotid artery accounts for 20–30% of all ischemic strokes [2]. Early diagnosis and adequate treatment with surgical removal of the atherosclerotic plaque (carotid endarterectomy) or stenting can prevent stroke [3, 4]. The principle indication for carotid revascularization is based on symptomatic status and degree of ipsilateral carotid artery stenosis. The degree of luminal stenosis is usually measured by conventional imaging modalities such as Doppler ultrasound. However, it has become increasingly clear that degree of luminal stenosis alone is not the best predictor of stroke risk and the morphology of the plaque plays a more crucial role. Research in carotid imaging is therefore focused on identifying characteristics that determine the unstable carotid plaque which have a high risk of future ipsilateral stroke. Based on histopathological studies, certain key plaque structural features such as a thin or ruptured fibrous cap (TRFC), a large lipid rich necrotic core (LRNC), intraplaque hemorrhage (IPH) or thrombus, inflammatory cells and intraplaque neovascularization (IPN), are associated with rupture –prone unstable plaques. New diagnostic methods which can identify unstable carotid plaques in vivo are therefore needed for a more precise targeting of prophylactic treatment and stroke prevention.

Atherosclerosis is a progressive immune-mediated inflammatory chronic disease of medium-and large-sized arteries, characterized by lipid accumulation and inflammation in the arterial wall [5, 6]. The presence of newly generated blood vessels arising from the adventitia within atherosclerotic lesions leading to intraplaque hemorrhage (IPH) plays a crucial role in the transition from a stable to an unstable plaque and is therefore associated with symptomatic carotid disease [7–9]. Visualization of adventitial vasa vasorum (VV) and intraplaque neovascularization has therefore recently emerged as a new possible surrogate marker for unstable atherosclerotic plaques [10, 11]. Conventional Doppler examinations filter out low-flow signals preventing the visualization of small blood vessels. Contrast-enhanced ultrasound (CEUS) has shown promise in the visualization of neovascularization [12], however, it requires intravenous injection of Ultrasound contrast agents (phospholipid-encapsulated sulphur hexafluoride microbubbles) with its associated risks [13, 14]. Superb microvascular imaging (SMI)(Canon Medical Systems Corporation Otawara, Japan) is a novel technique which can successfully depict microvascular blood flow signals without the use of contrast agents.

### Hypothesis

We postulate that carotid plaque neovascularization will be significantly more pronounced in plaques that exhibit progression in lesion area on ultrasound, and in plaques that cause ipsilateral vascular symptoms compared to asymptomatic plaques with stable morphology on ultrasound. If confirmed, this finding will strengthen the assumption that intraplaque neovascularization is associated with plaque instability. CEUS and SMI (non-invasive) are therefore bedside, safe and reliable methods to assess cardiovascular risk in these patients. Furthermore, our hypothesis is that IPN detected by SMI without the use of contrast agent is a predictor of ischemic stroke and cardiovascular mortality and that Shear Wave Elastography (SWE) provides additional information about plaque stiffness, atherosclerosis and stroke-risk.

### Aims of this study

The primary aim of this study is to compare intraplaque neovascularization and plaque tissue stiffness, assessed using advanced ultrasound methods such as SMI and SWE in patients with and without ipsilateral cerebrovascular symptoms. The second aim is to assess the level of agreement between structural plaque characteristics assessed using SMI, SWE with CEUS, GSM and carotid MRI, metabolic activity of plaques assessed by ^18^F-FDG PET/CT using histology as the gold standard. Findings from the methods mentioned above will be related to cerebrovascular symptoms, blood tests (cholesterol-tot, LDL, HDL, triglycerides, CRP, leukocytes, glucose, HbA1c) and other traditional cardiovascular risk factors at inclusion and at a 1-year follow-up.

## Design and methods

This study is a single center prospective observational study, designed to assess carotid plaque instability using multiparametric advanced ultrasound methods (SMI, SWE and CEUS) with molecular and radiological assessments. The histology of plaques removed at endarterectomy is the gold standard.

### Carotid MRI

Recent developments in magnetic resonance imaging (MRI) technology have shown promise with regard the identification of high-risk plaque characteristics and the accurate distinction between specific histological subtypes of carotid plaque characteristics to stratify risk of future stroke or transient ischemic attack (TIA). Gupta et al. [15] in a review of 9 MRI studies concluded that MRI characterization of specific plaque elements such as a large lipid rich necrotic core (LRNC), thin or ruptured fibrous cap (TRFC) and intraplaque hemorrhage (IPH) could provide additional measures of stroke risk not provided by static measurement of luminal stenosis. Semi-automated segmentation of carotid MRI can accurately measure LRNC size, which may be of help in the detection of vulnerable carotid plaque and assessment of stroke risk [16].

### PET imaging with 2-deoxy-2-(^18^F) fluoro-D-glucose (F^18^-FDG)

Positron emission tomography (PET) allows a direct assessment of the biological processes in a plaque. This is not possible using other imaging modalities, which provide information regarding luminal encroachment by the plaques and/or the structural features. Atherosclerotic disease is driven by a dynamic biological process (inflammation as a key component) and imaging plaque biology in addition to plaque structure may therefore provide important information. PET scanning with ^18^ F Fluorodeoxyglucose (^18^FFDG-PET) is a molecular imaging modality that is combined with CT or MRI scanning for both anatomical localization and attenuation correction. FDG is injected intravenously and is partially metabolized through glycolysis within the atherosclerotic plaque which serves as a marker of plaque inflammation and hypoxia. A major advantage of PET is its very high sensitivity, allowing Pico molar tracer concentrations to be detected that can be used to quantify the biological processes of interest. The conventional quantifying methods of radiotracer activity are the standardized uptake value (SUV) and tissue to background ratio (TBR). SUV may be further analyzed as SUV_max_ (highest tissue radiotracer concentration in a ROI) and SUV_mean._ (mean tissue radiotracer concentration within a whole ROI).

### Superb microvascular imaging (SMI)

Normal Doppler based methods have neither the spatial resolution nor the ability to assess blood flow in plaque neovascularization which consists of very small vessels with low blood flow velocities in [17]. This is due to the application of a motion wall filter when using Doppler techniques to remove wall motion artefacts and clutter. However, during this process, low velocity blood signals are also removed. SMI is a unique ultrasound technique developed by Canon (Canon Medical Systems, Aplio 300 US system) to overcome the limitations of conventional Doppler techniques. SMI employs an exclusive algorithm which does not filter low flow velocities and distinguishes true low-velocity flow from clutter. As a result SMI allows the visualization of minute vessels with low velocity flow signals without the use of contrast agent [18]. SMI has significant advantages in low-flow imaging including visualization of minute vessels, less motion artifacts, increased sensitivity with the use of CEUS, and high frame rates. SMI is potentially an inexpensive, bedside, safe, non-invasive method for stroke risk determination in clinical practice.

### Shear wave Elastography (SWE)

Shear Wave Elastography is an emerging ultrasound technique which exploits acoustic radiation forces to generate shear wave propagation in tissue, enabling the assessment of tissue stiffness by quantifying the Young’s modulus (YM) [19, 20]. Recent research suggests that a plaque ruptures when the peak stress within the fibrous cap exceeds a certain level [20]. Knowledge about the stress distribution within the fibrous cap could predict the risk of plaque rupture. SWE has been studied in liver, breast, and thyroid as well as prostate. One recent study of 25 plaques (of 25 plaques 9 were classified as unstable) by J. W Garrard [19] demonstrated that SWE was able to identify plaques with features of vulnerability, and that SWE provides additional information related to plaque tissue characteristics such as the presence of intraplaque hemorrhage. The mean YM in unstable plaques was 29 kilopascal (Kpa) lower than in stable plaques [19]. Shear wave elastography is considered less operator dependent, and with better reproducibility than earlier ultrasound elastography techniques that are based on operator compression of the tissue to induce a transient stress and assess tissue deformation. The use of shear waves to quantify elasticity was first proposed by Sarvazyan et al. in 1998 [21] as a novel technique that enables quantification of Young’s modulus. The speed of propagation of the shear waves is dependent on the local density and the elastic modulus of the tissue. YM is typically estimated using the equation; YM = 3ρc2, where ρ is the density of tissue, and c is the shear wave propagation velocity.

### Statistical assessments

SPSS for Windows statistical software (version 25.0) will be used for data analyses. Chi-square test will be used to test the relationship between the categorical variables and Mann-Whitney U test to compare the non-parametric categorical variables with continuous variables. Coefficients of correlation will be calculated by the Spearman rho correlation. All statistical results will be considered significant when *p* < 0.05. For the determination of interrater variability, Cohen’s kappa will be used to measure agreement between the two different ultrasound examiners using the established grading of agreement: < 0 (no agreement), 0 to 0.2 (poor), 0.21 to 0.4 (fair), 0.41 to 0.61 (moderate), 0.61–0.80 (substantial), 0.81 to 1.0 (nearly perfect). The receiver operating characteristic (ROC) curves will be used to evaluate the accuracy of SMI and CEUS in predicting microvessels observed at histology. The analysis for sample size estimation are based on existing data from our recently published study [22]. In this dataset mean SMI count in the asymptomatic group was 1.8 compared to 3.0 in the symptomatic group with an expected difference in mean of 1.2. The common standard deviation was 2.0 in both groups. Calculation for Shear wave elastography (SWE) was based on J. W Garrard who reported mean young’s modulus (YM) using SWE in unstable plaques to be 50.0 kPa compared to 79.1 kPa in stable plaques with an expected difference in mean of 29 kPa [19]. With an alpha = .05 and power = 0.80, we calculated that we need to include 90 patients in total, 45 in each group to detect a statistical significant difference between the two groups on SMI ultrasound, and 21 patients in each group for SWE ultrasound.

### Studies

#### 1.A SMI in symptomatic vs asymptomatic

The correlation between SMI assessed neovascularization and degree of stenosis in symptomatic patients with > 50% carotid stenosis versus asymptomatic patients with > 50% carotid stenosis.

### Patient inclusion and exclusion criteria

45 consecutive patients > 18 years old with asymptomatic carotid stenosis > 50%, referred to our cerebrovascular lab for routine ultrasound examination of their carotid lesion and 45 patients with symptomatic carotid stenosis > 50% referred to cerebrovascular lab for preoperative ultrasound prior to carotid endarterectomy (CEA) will be recruited in this study following informed written consent. Exclusion criteria are: right to left cardiac shunt, severe pulmonary hypertension (pulmonary artery pressure > 90 mmHg), uncontrolled systemic hypertension, adult respiratory distress syndrome, contrast agent allergy and other relevant contraindications. All patients on inclusion will undergo: 1) Clinical and neurological evaluations 2) Blood tests, 3) Superb Microvascular imaging (SMI) and Contrast enhanced ultrasound (CEUS) 4) Shear wave Elastography (SWE) 5) Carotid MRI 6) PET/CT 7) DW-MRI 8) Histological assessments after the removal of plaque at CEA.

#### 1.B SMI and vascular events. Asymptomatic patient’s follow-up at 12 months

1) Clinical neurological evaluations 2) Blood tests 3) Superb Microvascular imaging (SMI) and Contrast enhanced ultrasound (CEUS) 4) Shear wave Elastography (SWE) 5) Carotid MRI 6) PET/CT 7) DW-MRI.

The correlation between SMI assessed neovascularization and clinical symptoms will be measured. The end points are ipsilateral cerebrovascular events (stroke / TIA), vascular mortality (stroke, myocardial infarction), vascular interventions (carotid artery surgery or stenting, coronary bypass surgery or stenting,) after 1 year. Findings will be related to the extent of neovascularization detected at inclusion and at 1-year-follow-up and 1. The progression of neovascularization during 1-year follow up, 2. The progression in lesion area and degree of stenosis assessed with annual ultrasound. In addition, the grade of stenosis will be quantified as a categorical variable by defining change in stenosis grade over time (30–49%, 50–69%, 70–99, 100%) (Stable stenosis or increase in 1, 2, or 3 categories of 2 years).

#### 2.A SWE, GSM in symptomatic vs asymptomatic

This study will use the same patient pool described in study 1 undergoing the previously described procedural examinations upon inclusion and at one-year follow up. Quantification of stiffness/elasticity will be performed and compared to plaque echogenicity measured as GSM (grayscale median). These results will be correlated to the histology of the carotid plaques after endarterectomy, risk markers in blood (lipids, HbA1c, CRP, leukocytes), other cardiovascular risk factors (hypertension, diabetes, nicotine use, BMI, alcohol use) and cerebrovascular symptoms.

#### 2.B SWE, GSM and vascular event. Asymptomatic patients follow up at 12 months

Plaque stiffness measurements of YM assessed by SWE and plaque echogenicity measured as GSM (grey scale median) will be correlated with clinical symptoms. The end points are ipsilateral cerebrovascular events (stroke / TIA), vascular mortality (stroke, myocardial infarction), vascular intervention (carotid artery surgery or stenting, coronary bypass surgery or stenting) after 1 year. Findings will be related to the stiffness measurements detected at inclusion and at 1-year-follow-up.

### Study performance

#### I ultrasound of the precerebral arteries

Imaging will be performed with a Canon ultrasound machine (Canon Medical Systems, Aplio 300 US system); using a 7 L probe for both standard and contrast enhanced ultrasound as well as superb microvascular imaging (SMI) and a 10 L probe for shear wave elastography (SWE).

### A) Standard ultrasound

After identification of the common carotid artery (CCA), carotid artery bifurcation (BIF), internal carotid artery (ICA) bilaterally by B-mode ultrasound, color Doppler and pulsed-wave Doppler, the following assessments will be registered: Intima-media thickness (IMT) measurements of proximal and distal CCA. Detection and localization of plaques with registration of length and width. Determination of plaque echogenicity (hypoechoic, predominantly hypoechoic, predominantly hyperechoic, hyperechoic) [23, 24]. Fibrous cap surface classification into: regular, irregular or ulcerated. Peak systolic velocity (PSV) measurements in CCA, ICA and grading of stenosis will be carried out based on velocities according to the consensus criteria of the Society of Radiologists in Ultrasound [25]. If needed additional end diastolic velocity (EDV) measurement and ICA/CCA ratio. The examination will be digitally stored for later review.

### B) SWE

An inbuilt software is used to quantify YM as absolute tissue stiffness in Kpa. When chosen on the Canon ultrasound unit, this SWE specific software provides a real-time elastography box which is representative of elasticity (Kpa) or speed (m/s) by means of a colorimetric map. This software also shows the shear wave propagation map in form of wave-front lines as quality control. YM measurements of a given ROI within the elastography box when these propagation lines are parallel to each other is the most reliable, and if these lines are absent or distorted, measurement may need to be repeated. ROI’s will be placed as follows:

1) Manually drawn ROI to include the entire plaque and determine average, SD and range YM values via a work sheet on the Canon system for the whole plaque.

2) Place 3 circular ROIs in hard/high stress zone (red) within the plaque and 3 ROIs in the soft/low stress zone (blue) within the previously marked plaque and determine average, SD and YM range values for each ROI.

### C) Advanced ultrasound SMI, CEUS

#### Part 1. Semi-quantitative SMI

After choosing the inbuilt monochrome SMI (mSMI) software, the SMI specific ROI box will be positioned to portray the entire plaque. Other settings are a mechanical index of 1.5, frame rate of 50–60 frame per seconds (fps), dynamic range of 55–65 dB and velocity less than 2.0 cm/s. Plaques will be observed for 2 min and video images will be stored in the scanners hard drive. The intraplaque microvascular flow (IMVF) signals will firstly be categorized on a visual scale as follows: Grade 0: no IMVF within the plaque or IMVF confined to the adjacent adventitia, Grade 1: moving IMVF confined to the adventitial side, Grade 2: moving IMVF at the plaque shoulder, Grade3: IMVF moving to the plaque core, Grade 4: extensive IMVF. Secondly, a visual count of IMVF signal will be carried out and number of neo-vessels in a two-minute video clip will be counted.

#### Part 2. Semi-quantitative and quantitative analysis of CEUS

Preset real-time contrast-specific image settings (pulse inversion, MI 0.12) will be chosen from the scanner for optimizing images and to avoid destruction of contrast microbubbles. SonoVue (Bracco SpA, Milan, Italy), a microbubble contrast agent containing sulfur hexafluoride gas abilized with phospholipids (2.5 ml) will be injected as an intravenous bolus followed by 5 ml saline for semi-quantitative and quantitative analysis. Data registration starts upon the arrival of contrast material to the carotid artery bifurcation. Video clips will be stored as RAW data.

a**.** Semiquantitativ analysis:

The contrast enhancement in each plaque will be categorized on a visual scale as follows: Grade 0: no bubbles within the plaque or bubbles confined to the adjacent adventitia, Grade 1: moving bubbles confined to the adventitial side, Grade 2: moving bubbles at the plaque shoulder, Grade 3: bubbles moving to the plaque core, Grade 4: extensive intraplaque enhancement.

b**.** Quantitative analysis: Quantitative assessment of plaque contrast enhancement will be performed on RAW data off-line by plotting time-intensity curve (TIC) analysis using built-in quantification software (Canon, medical systems). A ROI will be drawn manually to include the entire plaque and a second circular ROI will be placed in the lumen of the artery as reference. Motion tracking and curve fitting will be applied to the TIC and the TIC derived peak intensity (PI) value will be obtained. PI in 10E-5 AU (arbitrary unit) is a value that is correlated with the blood vessel density in a given volume of tissue, expressing the maximum intensity relative to baseline of TIC.

### II blood sampling

Venipuncture of a forearm vein will be performed on the same day as the ultrasound examination except for those patients scheduled for carotid endarterectomy (CEA) where the blood test will be performed within 2 days prior to CEA.

### Blood tests

Plasma: 2 EDTA tubes (6 ml) will be placed on ice/cool water. The tubes will be centrifuged within 30 min at 3200 rpm. Plasma will be stored in aliquots tubes (Nunc) at 80 °C.

Serum: 1 serum tube (6 ml) will be stored in room temperature (max 2 h). After full coagulation, the tube will be centrifuged for 10 min at 3200 rpm before it is stored in aliquots tubes (Nunc) at 80 °C. Values of white blood cells, CRP, ESR, glucose, HbA1c, cholesterol, HDL, LDL and TG will be determined. Biobank blood tests will be used for determination of inflammation markers (Matrix metalloproteinase 7/ MMP-7, interleukin-23 / IL23, visfatin.

### III carotid MRI

The carotid arteries will be imaged using a 3 T whole-body scanner (Achieva, Philips Healthcare, Best, The Netherlands) equipped with an 8-channel carotid coil (Philips/Shanghai Chenguang Medical Technologies, Shanghai China). For each scan, the location of the carotid bifurcation will be determined using a 3D time-of-flight angiographic sequence, followed by 8 continuous slices using proton density, high resolution 3D time-of-flight T2 weighted and T1 weighted images.

Custom software (VP Diagnostics, Seattle, USA) will be used for the automatic analysis of the MRI examinations for plaque content, including neovascularization.

### IV 18F-FDG PET co-registered with enhanced CT

A subgroup of the study population will be examined with a hybrid PET/CT scanner (Siemens Biograph 64, Siemens Medical Systems, Erlangen, Germany). After an overnight fast (minimum six-hours), an ^18^F-FDG PET/CT will be performed from the base of the skull to the aortic arch. Approximately 90 min after the injection of 5Mbq/kg ^18^FFDG blood glucose levels will be measured. A CT without contrast for attenuation correction will be performed immediately before the PET scan with the patient in the same position. A contrast-enhanced CT of the carotid arteries will also be performed on those patients that do not have a recent CT angiography available. The contrast-enhanced CT will be used for localizing the carotid artery plaque. A specialist in nuclear medicine blinded for patient data will place the ROI. The contrast-enhanced CT angiography is used as a guide for drawing the ROI on the PET slice (fused with non-contrast CT). ROIs covering the whole plaque including vessel wall thickening and the lumen contrast-filling defect are drawn on each axial slice from the most cranial to the most caudal slice of the plaque.

### DWI-MRI

Patients will undergo cerebral diffusion-weighted imaging MRI (DWI-MRI) a 3 T whole-body scanner (Achieva, Philips Healthcare, Best, The Netherlands) on inclusion and at 1- year follow-up. DWI sequences will be used at each scan to detect new ischemic brain lesions. Detection of ischemic brain lesions/infarct during the follow-up time will strengthen the assumption of an unstable plaque. A neuroradiologist, blinded to clinical status and findings, will rate the diffusion-weighted trace images for the absence or presence of acute ischemic parenchymal damage. Positive, lesions will be quantified by using the following scoring system: number of lesions, location of lesions, lesion sizes (categorized into lesions < 5 mm, 5–10 mm, or > 10 mm), and total lesion volume (milliliters). Locations will be described to determine the vascular territories (anterior or posterior circulation), the side (ipsilateral or contralateral to the ICA stenosis), and the distribution (cortical, subcortical, or deep areas) [26]. The results from DWI-MRI will be correlated to degree of neovascularization obtained by the advanced ultrasound methods: SMI, CEUS and SWE.

### IV histological assessment

The plaques will be removed en bloc (intact) at endarterectomy (CEA), fixed in 4% formaldehyde, decalcified in ethylenediaminetetraacetic acid or 17% formic acid and cut into 2–3 mm slices. After dehydration the slices will be embedded in paraffin. Histological sections, measuring 5 μm, will be cut at and stained with hematoxylin and eosin. Plaques will be assessed by an experienced pathologist blinded for the clinical, carotid MRI, PET/CT and ultrasound findings and a research physician. In each section plaque area will be calculated based on measurements in a microscope with an ocular with micrometer scale. Areas with inflammation, granulation tissue, fat, fibrosis and calcification will be estimated as percentages of the plaque area. The percentages of the different components in a plaque will be calculated as the total area of each component in all sections from the plaque divided by the total plaque area. In each plaque-section number and diameters of vessels with a lumen diameter of 0.01 mm or greater will be measured. As an estimate of neovascularization of the plaque the sum of vessel lumens in all sections from the plaque will be divided by the total plaque area. The advanced Ultrasound (SMI, SWE and CEUS) findings will be correlated to the histology findings [27].

### V study population

Patients > 18 years referred to our ultrasound lab at the neurological outpatient clinic (Oslo University Hospital, Rikshospitalet) before endarterectomy or for routine ultrasound control, fulfilling study inclusion criteria will consecutively be asked to participate.

## Discussion

In spite of the fact that several characteristic features of unstable carotid plaques have been described in the past decade, no singular imaging modality has been demonstrated capable of identifying atherosclerotic plaques instability and the risk of rupture and consequent stroke. Pathological plaque angiogenesis, which is proliferation of new immature capillaries originating from adventitial vasa vasorum (VV) to the extent of plaque thickness, so-called intraplaque neovascularization (IPN) has been the focus of many recent studies. This growing interest in IPN is primarily due to the challenges associated with detection of microvascular blood flow by conventional ultrasonography methods and the fact that IPN detection in vivo can represent a step forward in diagnosis and follow-up of atherosclerosis burden. In healthy arteries, vessel wall is perfused and nourished by adventitial vasa vasorum penetrating only the adventitia and outer media [28]. Vasa vasorum is also involved in the repair of vascular damage by providing an increased supply of oxygen and nutrients. Upon progression of an atherosclerotic lesion, the adventitial VV responds to the hypoxia and increased metabolic demand of inflammatory cells by proliferating from the adventitia (less often from the main vessel lumen) to the full thickness of the media and intima of vascular wall and towards the lumen. This results in a disorganized and immature network of intraplaque neovessels. Endothelial cells in plaque with neovessels express more cell adhesion molecules than those in the main arterial lumen, which facilitate the further recruitment of inflammatory cells into the plaque [29]. Additionally, these microvessels are immature and fragile which have poorly established endothelial junction with incomplete pericytes coverage making them prone to rupture and hemorrhage. This promotes plaque instability and represents an important source of free cholesterol from red blood cells membranes, with consequent macrophage infiltration and necrotic core enlargement [10, 11]. CEUS and SMI have shown great promise in the visualization of intraplaque neovascularization [12, 14, 30–35], and studies have reported good correlation between IPN assessment by CEUS and micro-vessel density at histology [12, 36]. Previous studies also demonstrated a good consistency between assessment of IPN by CEUS and SMI. However, these studies lack histological validation and are limited by small sample size. To our knowledge only Zhang et al. [34] have combined assessment of IPN by CEUS and SMI with histological assessment using CD34 staining demonstrating a good correlation between the two methods. Unfortunately, in the study, the period between the ultrasound examination and histological assessment was not given and changes in the degree of IPN can therefore not be excluded. Determining the degree of intraplaque neovascularization reliably, could potentially serve as a new imaging marker for stroke risk stratification and preventive treatment decisions. Another potential marker of plaque instability is the measurement of stress distribution within the plaque. SWE enables the assessment of tissue stiffness by quantifying the elastic modulus or Young’s modulus (YM) providing valuable information about composition of the plaque. Previous studies have shown that a lower mean YM associated with focal neurological symptoms: transient ischemic attack (TIA), transient mononuclear blindness or stroke [19]. Additionally, significantly lower YM has been observed in plaques where intra-plaque hemorrhage or thrombus was present, and in plaques with increasing numbers of foam cells. SWE provides quantitative measurements of tissue stiffness by measuring the velocity of propagation of a shear-wave in tissues. Depending on tissue stiffness, shear waves travel at varying speed. The propagation speed of the shear waves directly correlates with tissue stiffness. SWE is less operator dependent compared to earlier elastography methods which are susceptible to the manner of manual compression by different operators, hence the poor reproducibility. Multiparametric ultrasound evaluations of atherosclerotic carotid plaques with the use of advanced ultrasound methods and conventional ultrasound may represent the future of bed side and non-invasive identification of unstable carotid plaque before the development of symptoms or catastrophic ischemic stroke. To our knowledge no other study has combined advanced new ultrasound technologies (SMI, CEUS, SWE) with other modalities such as carotid MR and PET/CT with histological validations as reference method. Results from this study may also pave the way towards developing imaging biomarkers for unstable carotid plaques and plaque progression indicating which modality provides the most complete information to guide individual stroke risk stratification and prophylactic treatment.

## Data Availability

Not applicable, the manuscript does not include any data.

## Abbreviations

CEA: Carotid endarterectomy
CEUS: Contrast enhanced ultrasound
CRP: C reactive protein
DSA: Intra-arterial digital subtraction angiography
DWI-MRI: Diffusion-weighted imaging MRI
Fps: Frame per second
GSM: Plaque gray-scale-median
IL23: Interleukin-23
IMT: Intima –media thickness
IPH: Intraplaque hemorrhage
IPN: Intraplaque neovascularization
Kpa: Kilopascal
LRNC: large lipid rich necrotic core
MMP-7: Matrix metalloproteinase 7
MRI: Magnetic resonance imaging
PET F18-FDG: Positron emission tomography, 2-deoxy-2-(18F) fluoro-D-glucose
ROI: Region of interest
SMI: Superb microvascular imaging,
SUV: Standardized uptake value
SWE: Shear Wave Elastography
TBR: Tissue to background ratio
TIA: Transient ischemic attack
TRFC: Thin or ruptured fibrous cap
VV: Arterial vasa vasorum
YM: Young’s modulus
